# (–)-Catechin-7-*O*-β-d-Apiofuranoside Inhibits Hepatic Stellate Cell Activation by Suppressing the STAT3 Signaling Pathway

**DOI:** 10.3390/cells9010030

**Published:** 2019-12-20

**Authors:** Yong Joo Park, Dong Min Kim, Mi Ho Jeong, Jae Sik Yu, Hae Min So, In Jae Bang, Ha Ryong Kim, Seung-Hwan Kwon, Ki Hyun Kim, Kyu Hyuck Chung

**Affiliations:** 1 School of Pharmacy, Sungkyunkwan University, Suwon 16419, Korea; pyj084@msn.com (Y.J.P.); kdm9947@gmail.com (D.M.K.); algh8906@naver.com (M.H.J.); jsyu@bu.edu (J.S.Y.); haemi9312@gmail.com (H.M.S.);; 2 College of Pharmacy, Daegu Catholic University, Gyeongsan 38430, Korea; kimhr@cu.ac.rk; 3 Neuroregeneration and Stem Cell Programs, Institute for Cell Engineering, The Johns Hopkins University School of Medicine, Baltimore, MD 21205, USA.; skwon28@jhmi.edu; 4 Department of Neurology, The Johns Hopkins University School of Medicine, Baltimore, MD 21205, USA

**Keywords:** (–)-Catechin-7-*O*-β-d-apiofuranoside, *Ulmus davidiana* var. *japonica*, hepatic stellate cells, STAT3, hepatic fibrosis

## Abstract

Hepatic fibrosis is characterized by the abnormal deposition of extracellular matrix (ECM) proteins. During hepatic fibrogenesis, hepatic stellate cell (HSC) activation followed by chronic injuries is considered a key event in fibrogenesis, and activated HSCs are known to comprise approximately 90% of ECM-producing myofibroblasts. Here, we demonstrated that (–)-catechin-7-*O*-β-d-apiofuranoside (C7A) significantly inhibited HSC activation via blocking the signal transducer and activator of transcription 3 (STAT3) signaling pathway. This is the first study to show the hepatic protective effects of C7A with possible mechanisms in vitro and in vivo. In our bioactivity screening, we figured out that the EtOH extract of *Ulmus davidiana* var. *japonica* root barks, which have been used as a Korean traditional medicine, inhibited collagen synthesis in HSCs. Four catechins isolated from the EtOAc fraction of the EtOH extract were compared with each other in terms of reduction in collagen, which is considered as a marker of hepatic protective effects, and C7A showed the strongest inhibitory effects on HSC activation in protein and qPCR analyses. As a possible mechanism, we investigated the effects of C7A on the STAT3 signaling pathway, which is known to activate HSCs. We found that C7A inhibited phosphorylation of STAT3 and translocation of STAT3 to nucleus. C7A also inhibited expressions of *MMP-2* and *MMP-9*, which are downstream genes of STAT3 signaling. Anti-fibrotic effects of C7A were evaluated in a thioacetamide (TAA)-induced liver fibrosis model, which indicated that C7A significantly inhibited ECM deposition through inhibiting STAT3 signaling. C7A decreased serum levels of aspartate amino transferase and alanine transaminase, which were markedly increased by TAA injection. Moreover, ECM-associated proteins and mRNA expression were strongly suppressed by C7A. Our study provides the experimental evidence that C7A has inhibitory effects on HSC activation after live injury and has preventive and therapeutic potentials for the management of hepatic fibrosis.

## 1. Introduction

Hepatic fibrosis, which has characteristics of excessive deposition of extra cellular matrix (ECM), occurs by various chronic liver injuries, such as from chemicals, viral infection, and alcoholic or nonalcoholic hepatic steatosis [1]. Although the pathogenesis of hepatic fibrosis is not revealed clearly, hepatic stellate cells (HSCs) are believed to be a major role in the development of hepatic fibrosis and have received a lot of attention. Quiescent HSCs are located in the sub-endothelial space of Disse and have cytoplasmic lipid droplets as a storage depot for vitamin A [2]. When injuries occur, quiescent HSCs undergo transdifferentiation from vitamin A storage cells to myofibroblast-like cells. Activated HSCs express α-smooth muscle actin (α-SMA) and release excessive ECMs, including collagens and cellular fibronectin, which are predominant in progressive hepatic fibrosis [3,4,5,6].

Since HSCs are an important therapeutic target in hepatic fibrosis pathogenesis, greater advances have been made in understanding the molecular mechanisms associated with HSC activation. Now, inhibition and reversion of activated HSCs are proposed as potential therapeutic strategies for hepatic fibrosis [7,8,9]. The signal transducer and activator of transcription 3 (STAT3) is a transcription factor involved in proliferation and activation of HSCs [10,11]. In response to cytokines and growth factors after liver injury, STAT3 is phosphorylated by cytoplasmic tyrosine kinases, Janus kinases (JAKs), followed by dimerization and translocation to the nucleus to transcript target genes [12,13]. Recent studies reported that inhibition of STAT3 can be a therapeutic target to inhibit HSC activation [14,15]. TGF-β1 is a well-known pivotal cytokine in development of hepatic fibrosis through SMAD and non-SMAD pathways [16,17]. It was reported that both STAT3 and TGF-β1 signaling pathways coordinate to activate HSCs, especially, TGF-β1 can promote the STAT3 pathway through the non-SMAD JAK/STAT3 pathway [17].

As part of our continuing projects to explore biologically active, unique natural products from diverse natural sources [18,19,20,21,22], we recently took note of *Ulmus davidiana* var. *japonica*, the root barks of which have been used as a Korean traditional medicine for various therapeutic purposes including gastroenteric disorders and inflammatory disorders [23,24,25,26]. Previous studies of pharmacological investigations on *U. davidiana* var. *japonica* have reported that extracts of this plant have shown potency antioxidative, anti-inflammatory, and anticancer activities [27,28,29]. Phytochemical investigation of *U. davidiana* var. *japonica* root bark has demonstrated the existence of diverse metabolites, flavonoids, triterpene esters, sesquiterepenes, lignans, and neolignan glycosides [25,30,31,32,33]. In our bioactivity screening, we figured out that the EtOH extract of *U. davidiana* var. *japonica* root barks inhibited HSC activation. Solvent partitioning of the EtOH extract afforded four main fractions, and among the fractions, the EtOAc-soluble fraction suppressed fibrotic effects in activated HSCs. In line with this evidence, we investigated potential metabolites associated with the inhibition of HSC activation from the EtOAc-soluble fraction for the treatment of hepatic fibrosis. Column chromatographic separation of the EtOAc fraction led to the isolation of four catechins (**1**–**4**), followed by HPLC purification.

In this study, a western blot analysis was conducted to evaluate the inhibitory effects of the isolated catechins on collagen synthesis through HSC activation. For establishing the underlying inhibition mechanism, we evaluated the effect of the most active compound, (–)-catechin-7-*O*-β-d-apiofuranoside (C7A), on the HSC activation marker, α-SMA, and collagen by western blot and qPCR analyses. We also investigated whether C7A activates molecular mechanism pathways associated with TGF-β1 and STAT3 using western blot and immunofluorescence analyses. Furthermore, anti-fibrotic effects of C7A were evaluated in a thioacetamide (TAA)-induced liver fibrosis model. Herein, we described in more detail the isolation of C7A, its inhibitory effects on HSCs via inhibiting STAT3 activation, and the anti-fibrotic effects of C7A in a mice model.

## 2. Materials and Methods

### 2.1. General Experimental Procedures

Optical rotations were calculated using a Jasco P-1020 polarimeter (Jasco, Easton, MD, USA). Electronic circular dichroism (ECD) spectra in MeOH were acquired in a quartz cuvette of 1 mm optical path length on a JASCO J-1500 spectropolarimeter (Tokyo, Japan). Ultraviolet (UV) spectra were acquired on an Agilent 8453 UV–visible spectrophotometer (Agilent Technologies, Santa Clara, CA, USA). NMR spectra were recorded on a Varian UNITY INOVA 700 NMR spectrometer operating at 800 MHz (^1^H) and 200 MHz (^13^C), with chemical shifts given in ppm (δ). Preparative high-performance liquid chromatography (HPLC) was performed on a Waters 1525 Binary HPLC pump with Waters 996 Photodiode Array Detector (Waters Corporation, Milford, CT, USA). Semi-preparative HPLC utilized a Shimadzu Prominence HPLC System with SPD-20A/20AV Series Prominence HPLC UV–Vis Detectors (Shimadzu, Tokyo, Japan). LC/MS analysis was conducted on an Agilent 1200 Series HPLC system (Agilent Technologies, Santa Clara, CA, USA) equipped with a diode array detector and a 6130 Series ESI mass spectrometer using an analytical Kinetex (4.6 × 100 mm, 3.5 μm). Silica gel 60 (Merck, 70–230 mesh and 230–400 mesh) and RP-C_18_ silica gel (Merck, 40–63 μm) were used for column chromatography. The packing material for molecular sieve column chromatography was Sephadex LH-20 (Pharmacia, Uppsala, Sweden). Diaion HP-20 (Mitsubishi Chemical, Tokyo, Japan) was also used for open-column chromatography. Precoated silica gel F_254_ plates and RP-18 F_254s_ plates (Merck, Darmstadt, Germany) were used for TLC. Spots were detected on TLC under UV light or by heating after spraying with anisaldehyde–sulfuric acid.

### 2.2. Plant Materials

The root barks of *U. davidiana* var. *japonica* (Rehder) Nakai were collected in Wonhwasan-ro, Jecheon-si, Chungcheongbuk-do, Korea, in 2016 and purchased by Donggwang General Corporation. The plant was authenticated by one of the authors (K. H. Kim). A voucher specimen (SKKU-NR 0401) of the plant has been deposited at the herbarium of the School of Pharmacy, Sungkyunkwan University, Suwon, Korea.

### 2.3. Extract and Isolation

The dried root barks of *U. davidiana* var. *japonica* (10 kg) were extracted with 50% aqueous EtOH (each 60 L × 2 d) at 70 ℃ and filtered. The filtrate was combined and concentrated under reduced pressure using a rotavapor to obtain crude EtOH extract (900 g). Part of the extract (300 g) was suspended in distilled water (800 mL) and successively solvent-partitioned with hexane, dichloromethane (CH_2_Cl_2_), ethyl acetate (EtOAc), and *n*-butanol (BuOH). This resulted in four different fractions of polarity: hexane-soluble (4.0 g), CH_2_Cl_2_-soluble (39.0 g), EtOAc-soluble (25.0 g), and BuOH-soluble fractions (81.0 g). With the guidance of bioactivity of the four fractions, the EtOAc-soluble fraction was selected for phytochemical investigation. The EtOAc-soluble fraction (25 g) was subjected to a Diaion HP-20 column in a MeOH gradient solvent system to yield six fractions (E0, E2, E4, E6, E8, and E10). Fraction E4 (4.5 g) was further subjected to silica gel column chromatography (200 g, eluted with CH_2_Cl_2_/MeOH (20:1→ 1:1), gradient system) to yield seven fractions (E4A–E4G). Fraction E4C (2.2 g) was separated by RP-C18 column chromatography with a gradient solvent system of MeOH–H_2_O (10–100% MeOH) to yield seven subfractions (E4C1–E4C7). Fraction E4C4 (1.7 g) was separated by preparative reversed-phase HPLC with a gradient solvent system of MeOH–H_2_O (10–80% MeOH) to give four fractions (E4C41–E4C44). Fraction E4C42 (0.4 g) was separated by semi-preparative HPLC (18% MeOH) to yield compounds **1** (*t*_R_ 46.0 min, 155.6 mg), **2** (*t*_R_ 37.5 min, 42.9 mg), and **3** (*t*_R_ 29.0 min, 57.0 mg). Fraction E4D (4.2 g) was separated on Sephadex LH-20 column using solvent system of MeOH–H_2_O (50% MeOH) to yield five subfractions (E4D1–E4D5). Fraction E4D2 (0.4 g) was purified using semi-preparative HPLC (18% MeOH) to furnish compound **4** (*t*_R_ 42.0 min, 30.5 mg).

### 2.4. Cell Lines and Culture

Human hepatic cell line LX-2 was obtained from the American Type Culture Collection (ATCC, Manassas, VA, USA). Cells were cultured in Dulbecco’s modified Eagle medium (Sigma, St. Louis, MO, USA) supplemented with 10% fetal bovine serum (Biotechnics Research Inc., Lake Forest, CA, USA), penicillin (100 units/mL), and streptomycin (100 ug/mL). Cells were maintained at 37 °C in atmosphere containing 95% air and 5% CO_2_ under saturated humidity.

### 2.5. Animal Experiment

All animal experiments were approved by the Sungkyunkwan University Animal Care Committee and conducted in accordance with the guidelines of the National Institutes of Health (SKKUIACUC2018-10-44-2). Male C57BL/6 mice (6 weeks old; 22–25 g) were purchased from Daehan BioLink (Chungbuk, Korea). Water and food were provided ad libitum. After a week of acclimatization, mice were randomly divided into four groups: (1) mice received vehicle (saline) and vehicle (saline), (2) mice received C7A and saline, (3) mice received saline and thioacetamide (TAA), and (4) mice received C7A and TAA. Mice were treated with TAA (150 mg/kg) through intraperitoneal injection (I.P.) three times per week for 6 weeks. On day 22, mice were treated with saline or C7A (40 mg/kg) through I.P. injection three times per week for 3 weeks. Mice were anaesthetized on day 43, and blood and liver tissue samples were collected for analysis.

### 2.6. Liver Histology and Blood Analysis

Liver tissues were fixed in 10% buffered formalin, embedded in paraffin, and cut into 4 µm sections. The sections were then stained with hematoxylin and eosin (H&E) and Masson’s trichrome. Aspartate aminotransferase (AST) and alanine transaminase (ALT) levels in mice serum were analysed as a measure of liver function (ChemOn Inc. Suwon, Korea).

### 2.7. Hydroxyproline Measurement

The collagen level in the liver tissue was determined by using a hydroxyproline assay (BioVision, K555-100, Milpitas, CA, USA) in accordance with the manufacturer’s protocol. Briefly, 10 mg of liver tissue was homogenized and hydrolyzed at 120 °C for 3 h in 6 N HCl. Then, the samples were centrifuged at 10,000× *g* for 5 min, and the supernatant was collected for hydroxyproline determination. The absorbance at 560 nm was measured using a microplate reader (Molecular Devices, Sunnyvale, CA, USA).

### 2.8. Cell Viability Assay

Cell viability was analyzed by WST-1assay (Roche, Mannheim, Germany). LX-2 cells were seeded onto 96-well plates at a density of 1 × 10^5^ and incubated for 24 h. Then, cells were treated with chemicals for 48 h. A WST-1 reagent was added to each well according to the manufacturer’s instruction and incubated within 30 min at 37 °C. Absorbance was measured at 440 nm and 690 nm using a microplate reader (Molecular Devices).

### 2.9. Comparative Quantitative Real-Time PCR (qPCR)

Total RNA was isolated using TRIzol reagent (Life Technologies, Grand Island, NY, USA) following the protocol provided by the manufacturer. RNA concentration was measured using BioDrop Duo (Biodrop, Cambridge, UK), and cDNA was synthesized by a High-Capacity cDNA Reverse Transcription System (Life Technologies). qPCR was performed in duplicate for each sample using SYBR^®^ Premix Ex Taq^TM^ (Life Technologies) and CFX96 Real-Time PCR System (Bio-Rad, Hercules, CA, USA). qPCR was performed using the primers listed in Table 1. Expression levels of mRNA were normalized to GAPDH.

### 2.10. Western Blot Analysis

LX-2 cells were seeded in a six-well plate at 5 × 10^4^ cells/well and incubated for 24 h. After TGF-β1 treatment for 48 h, chemicals were treated for 48 h. Cells were washed twice with PBS and lysed with radioimmunoprecipitation assay buffer (Thermo Scientific) with protease inhibitor cocktail (GenDEPOT, Barker, TX, USA), phosphate inhibitor (BioVision, Milpitas, CA, USA), and 0.1% SDS. The cell lysates were incubated on ice for 30 min and subsequently centrifuged at 13,000× *g* for 15 min at 4 °C. Pierce^TM^ BCA Protein Assay Kit (Pierce, Rockford, IL, USA) was used to quantify the protein concentration. Samples were denatured with buffer containing 2% SDS, 6% 2-mercaptoethanol, 40% glycerol, 0.004% bromophenol blue, and 0.06 M Tris–HCl at 90–100 °C for 6 min, then cooled at room temperature for 5 min. A sample of 15 ug of each protein was resolved in 10% or 8–16% gradient SDS-PAGE gel (Bio-Rad) and transferred to polyvinylidene difluoride (PVDF) membrane (Bio-Rad). The membrane was blocked with 5% skim milk in TBS-T at room temperature for 1 h and incubated with primary antibodies overnight at 4 °C. Then, the membranes were washed with TBS-T and incubated with secondary antibodies conjugated to horseradish peroxidase for 1 h. The protein bands were developed with enhanced chemiluminescence (ECL) reagents (Bio-Rad) using an automatic X-ray film processor (JPI Healthcare, Seoul, Korea). The densities of each band were normalized to those of the GAPDH band. Anti-fibronectin (ab2413), anti-collagen (ab138492), anti-alpha-SMA (ab5694, abcam), anti-MMP-2 (ab37150), anti-MMP-9 (ab38898, Abcam, Cambridge, MA, USA), anti-STAT3 (#9139), anti-p-STAT3 (#9145), anti-TIMP-1 (#8946), anti-TIMP-2 (#5738), anti-laminA/C (#2032), anti-CTGF (#86641, Cell signaling technology, Danvers, MA, USA), and anti-GAPDH (015-25473, Wako pure chemical industries, Osaka, Japan) were used in western blot analysis.

### 2.11. Preparation of Nuclear Extracts

LX-2 cells were washed with ice-cold PBS and then lysed with hypotonic buffer (10 mM HEPES (pH 7.9), 10 mM KCl, 1.5 mM MgCl_2_, 1 mM EDTA, 10 mM protease inhibitor cocktail, and 10 mM protein phosphatase inhibitors) containing 0.75% NP-40 on ice for 15 min. After centrifugation at 3000 rpm for 5 min at 4 °C, cell pellets were rinsed with hypotonic buffer and then resuspended in high-salt buffer (20 mM HEPES (pH7.9), 0.4M NaCl, 1 mM EDTA, glycerol 25%) at 4 °C for 15 min. Nuclear extracts were collected from supernatants by centrifugation at 13,000× *g* for 5 min at 4 °C.

### 2.12. Immunocytochemistry

LX-2 cells were plated on a 12-well glass slide plate. Cells were pretreated with TGF-β1 for 48 h followed by chemical exposure for another 48 h. Cells were washed with PBS, fixed with 4% paraformaldehyde, and permeabilized with 0.1% Triton X-100 in PBS. Slides were blocked with 5% bovine serum albumin in PBS for 1 h and then incubated with a primary p-STAT3 antibody for 1 h at 37 °C. After washing with PBS, the cells were incubated with secondary fluorescent antibody (goat anti-rabbit IgG FITC conjugates) for 1 h protected from the light. Cells were nuclear-stained by Prolong^TM^ Diamond Antifade Mountant with DAPI (Invitrogen, Carlsbad, CA USA), and images were captured using Zeiss LSM 700 Laser Confocal Microscope (Carl Zeiss, Jena, Germany).

### 2.13. Statistical Analysis

The data were analyzed using GraphPad Prism version 7.00 (GraphPad software Inc., San Diego, CA, USA) and Excel (Microsoft, Redmond, WA, USA). Each assay was performed a minimum of three times. The data from each assay were expressed as the mean ± standard deviation (SD). The differences between the groups were assessed by Duncan’s post hoc test after one-way analysis of variance (ANOVA). Statistical significance was accepted at *p* < 0.05.

## 3. Results

### 3.1. EtOH Extract of U. davidiana var. japonica and Its EtOAc-Soluble Fraction Suppress Collagen Synthesis in Activated HSCs

**Figure 1:**
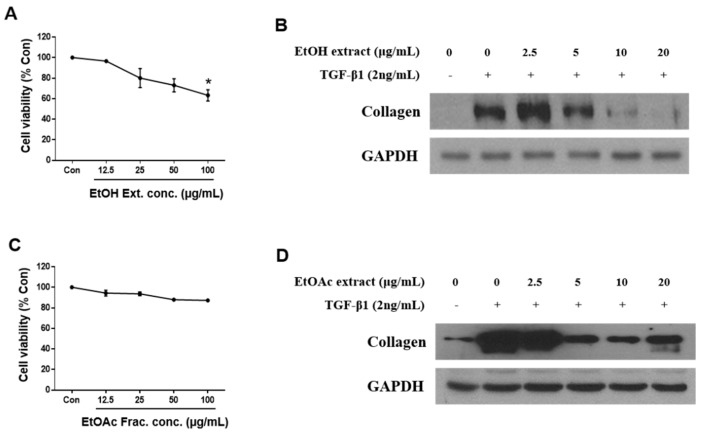
EtOH extract and EtOAc fraction of *Ulmus davidiana* var. *japonica* suppress the fibrotic effect in activated LX-2 cells. LX-2 cells were treated with EtOH extract or EtOAc fraction of *U. davidiana* var. *japonica* for 48 h after TGF-β1 induction for 48 h. Cytotoxicities of (**A**) EtOH extract and (**C**) EtOAc fraction in LX-2 cells were investigated by WST-1 assay after treatment for 48 h. The protein expression of collagen was analyzed by western blot assay in (**B**) EtOH extract and (**D**) EtOAc fraction treated groups. GAPDH was used as a loading control. Each experiment was repeated three times, and values represent mean ± S.D. * *p* < 0.05 compared with control.

The dried root barks of *U. davidiana* var. *japonica* were extracted with 50% aqueous EtOH at 70 °C, and the filtrate was concentrated under vacuum to obtain EtOH extract. Firstly, after treatment of the EtOH extract for 48 h, cytotoxicity was measured by WST-1 assay, which is based on the measuring mitochondrial dehydrogenase enzymes (Figure 1A). Cytotoxicity of the EtOH extract was represented dose dependently, and the appropriate concentration (2.5–20 μg/mL) was determined based on cytotoxicity to test the inhibitory effect on hepatic fibrosis. TGF-β1 was used to activate HSCs, and expression of collagen was analyzed as a hall marker of ECM deposition. As a result, the EtOH extract significantly suppressed collagen expression at the concentration of 5 μg/mL (Figure 1B). We then fractionated the EtOH extract into four main fractions, hexane, CH_2_Cl_2_, EtOAc, and *n*-BuOH-soluble fractions, and we examined which fraction was enriched with the active metabolites associated with inhibition of HSC activation. Among the fractions obtained, the EtOAc-soluble fraction showed the most potent inhibitory effects on HSC activation (data not shown). The EtOAc-soluble fraction also inhibited collagen synthesis in activated HSCs without cytotoxicity (Figure 1C,D).

### 3.2. Chemical Investigation of the EtOAc Fraction Led to the Isolation of Four Catechins

**Figure 2:**
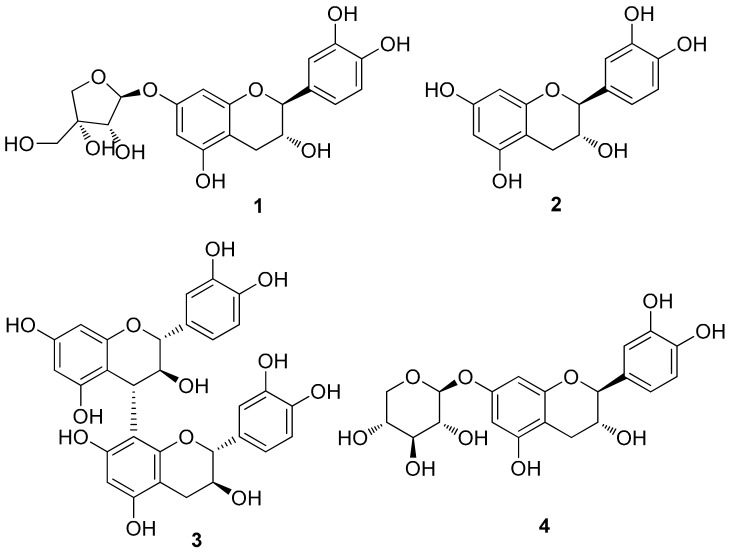
Chemical structures of catechins (**1**–**4**) isolated from *U. davidiana* var. *japonica*.

To identify the metabolites responsible for the observed activity of the EtOH extract, a chemical investigation was performed on the EtOAc-soluble fraction. Repeated column chromatography and HPLC purification led to the isolation of four catechins (**1**–**4**) (Figure 2). The isolated catechins were identified as (–)-catechin-7-*O*-β-d-apiofuranoside (**1**) [34], (–)-catechin (**2**) [35], procyanidin B3 (**3**) [36,37], and (–)-catechin-7-*O*-β-d-xylopyranoside (**4**) [38] by the comparing their NMR spectroscopic and physical data with those in the literature and by measuring their ECD data as well as LC/MS analysis.

### 3.3. C7A Inhibits the Fibrotic Effects in HSC Activation

**Figure 3:**
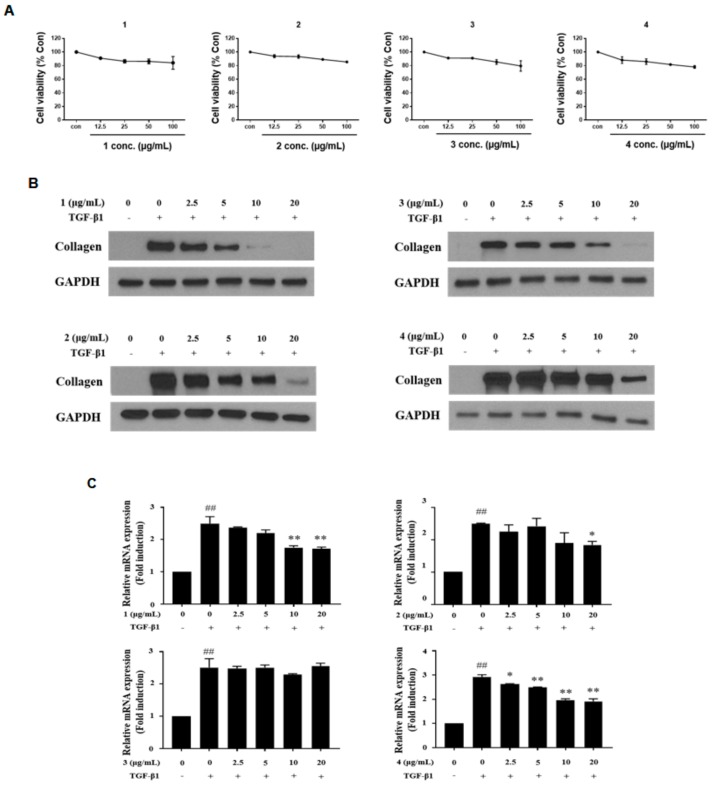
Antifibrotic effects of compounds (**1**–**4**) from EtOAc fraction in activated LX-2 cells. The **i**nhibitory effects of compounds (**1**–**4**) were tested by treating them for 48 h, respectively, after TGF-β1 induction for 48 h. (**A**) Cytotoxicity of compounds (**1**–**4**) in LX-2 cells was evaluated by WST-1 assay after treatment for 48 h. (**B**) The protein expressions of collagen were analyzed by western blot assay in each compound treated group. (**C**) Relative collagen mRNA expressions were analyzed by qPCR analysis. Each experiment was repeated three times, and values represent mean ± S.D. ## *p* < 0.01 compared with control, ** *p* < 0.01, * *p* < 0.05 compared with TGF-β1 treatment group.

To figure out the most potent active compound to exert antifibrotic effects in activated HSCs, the cytotoxicity of four catechins was tested by WST-1 assay after treatment of compounds **1**–**4** for 48 h (Figure 3A). As a result, all compounds showed dose-dependent cytotoxicity; however, no compounds showed significant cytotoxicity in the range of concentration 12.5–100 μg/mL. Then, compounds **1**–**4** (2.5–20 μg/mL) were treated for 48 h to evaluate antifibrotic effects in TGF-β1-activated LX-2 cells. All compounds inhibited collagen synthesis in HSCs, and intriguingly, compound **1** (C7A) showed the strongest effects on collagen synthesis in protein and qPCR analyses (Figure 3B,C). Collagen synthesis was significantly increased by TGF-β1 treatment; however, 10 μg/mL of C7A strongly suppressed collagen synthesis in activated HSCs.

**Figure 4:**
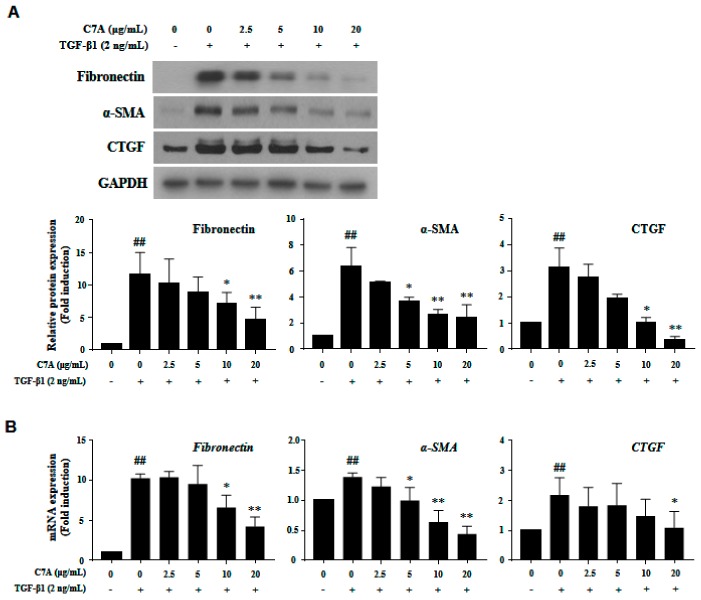
(–)-Catechin-7-*O*-β-d-apiofuranoside (C7A) suppresses the activation of LX-2 cells. Inhibitory effects of C7A on LX-2 cell activation were tested by treating it for 48 h after TGF-β1 induction for 48 h. (**A**) The protein expressions of fibronectin, α-SMA, and CTGF were analyzed by western blot assay, and relative protein expressions were obtained from Image J quantification values. (**B**) The mRNA expression levels of *fibronectin*, *α-SMA,* and *CTGF* were evaluated by qPCR analysis. Each experiment was repeated three times, and values represent mean ± S.D. ## *p* < 0.01 compared with control, ** *p* < 0.01, * *p* < 0.05 compared with TGF-β1 treatment group.

To explore the further effects of C7A on HSC activation, the protein and mRNA expressions of fibronectin, α-smooth muscle actin (α-SMA), and connective tissue growth factor (CTGF), which are hallmarks of HSC activation, were analyzed. As shown in Figure 4A, C7A significantly suppressed fibronectin, α-SMA, and CTGF protein expressions that were induced by TGF-β1 treatment. Moreover, the mRNA expression level of *fibronectin*, *α-SMA,* and *CTGF* were also decreased in a dose-dependent manner by C7A treatment (Figure 4B).

### 3.4. C7A Suppresses Fibrotic Response Through Regulating the STAT3 Signaling Pathway

**Figure 5:**
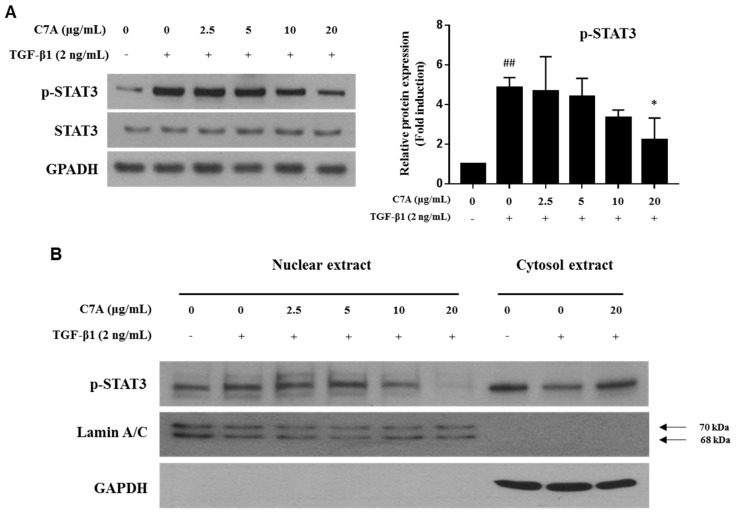

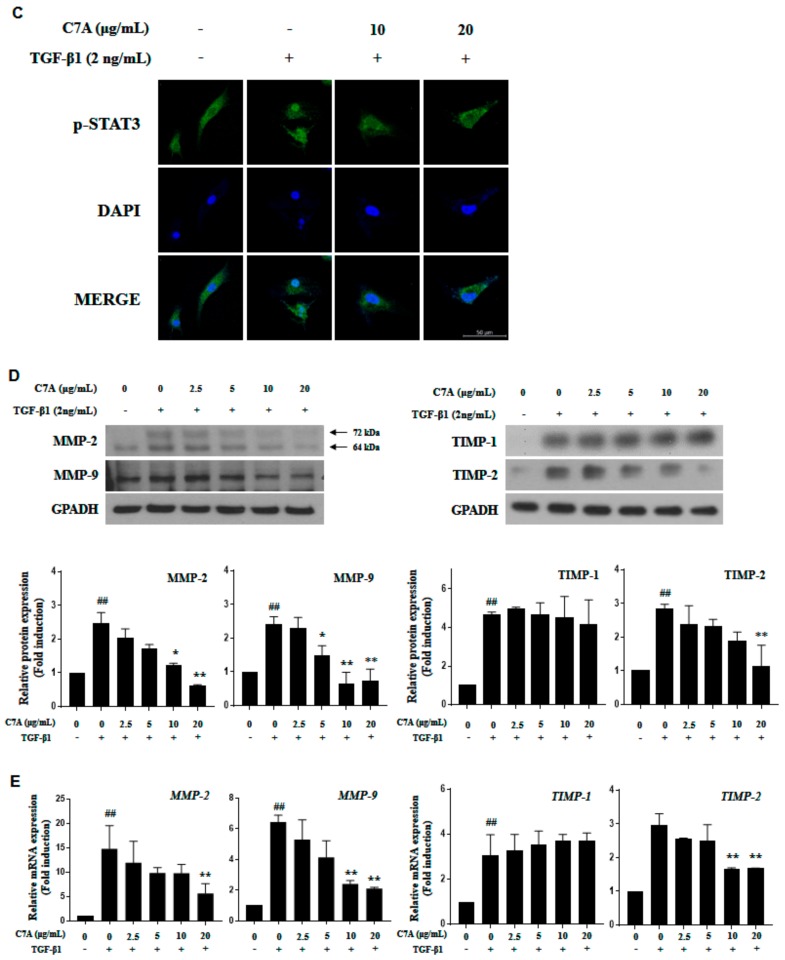
C7A suppresses STAT3 phosphorylation and translocation in TGF-β1-activated LX-2 cells. LX-2 cells were treated with C7A for 48 h after TGF-β1 induction for 48 h. (**A**) The expression levels of p-STAT3 and STAT3 were analyzed by western blot assay. (**B**) The expression levels of p-STAT3 were analyzed from nuclear and cytosolic protein fractions. Lamin A/C was used as a nuclear loading control. (**C**) p-STAT3 (green) localization in LX-2 cells was determined by confocal immunocytochemistry. The nuclei are counterstained with 4′,6-diamidino-2-phenylindole (DAPI) (Blue). The scale bars represent 50 µm. (**D**) The protein expression levels of MMP-2, MMP-9, TIMP-1, and TIMP-2 were measured by western blot assay. Densitometric analysis is expressed as mean ± SD intensity of optical density obtained by three independent experiments. (**E**) The mRNA expression levels of *MMP-2, MMP-9, TIMP-1,* and *TIMP-2* were evaluated by qPCR analysis. Each experiment was repeated three times, and values represent mean ± S.D. ## *p* < 0.01 compared with control, ** *p* < 0.01, * *p* < 0.05 compared with TGF-β1 treatment group.

The STAT3 pathway plays a critical role in hepatic fibrosis by regulating proliferation and activation of HSCs; therefore, inhibition of STAT3 has been considered as an important therapeutic target for hepatic fibrosis [14,15]. In this study, we found that C7A significantly inhibited TGF-β1-induced STAT3 phosphorylation (Figure 5A). To identify the mechanism of C7A on STAT3 inhibition, the p-STAT3 level in the nuclear fraction was analyzed. Translocation of p-STAT3 to the nucleus was induced by TGF-β1 treatment; however, C7A suppressed p-STAT3 translocation in a dose-dependent manner (Figure 5B). Nuclear translocation of p-STAT3 by TGF-β1 treatment was also observed by immunofluorescence assay, and it was blocked by C7A treatment (Figure 5C). Matrix metalloproteinase (MMP-2 and MMP-9) and tissue inhibitors of metalloproteinase (TIMP-1 and TIMP-2) are involved in cell migration and proliferation of activated HSCs. *MMPs* and *TIMPs* are known to be downstream genes of the STAT3 pathway and regulated by STAT3 activation [39,40]. TIMPs are associated with the regulation of MMP activities, and particularly, TIMP-1 and TIMP-2 are reported to be highly expressed in human liver fibrosis [41,42,43]. As regulators of MMPs, TIMP-1 can form a complex with pro-MMP-9, while both TIMP-2 and TIMP-4 can bind to the pro-MMP-2 [44,45]. To explore the effects on STAT3 inhibition by C7A, protein expressions of MMP-2, MMP-9, TIMP-1, and TIMP-2 were measured. As a result of C7A treatment in activated HSCs, C7A significantly down-regulated the protein expressions of MMP-2, MMP-9, and TIMP-2, but not TIMP-1 (Figure 5D). In addition, the mRNA expression levels of *MMP-2*, *MMP-9,* and *TIMP-2* were also significantly decreased by C7A treatment, but not TIMP-1 (Figure 5E).

### 3.5. C7A Attenuated TAA-Induced Chronic Liver Fibrosis

**Figure 6:**
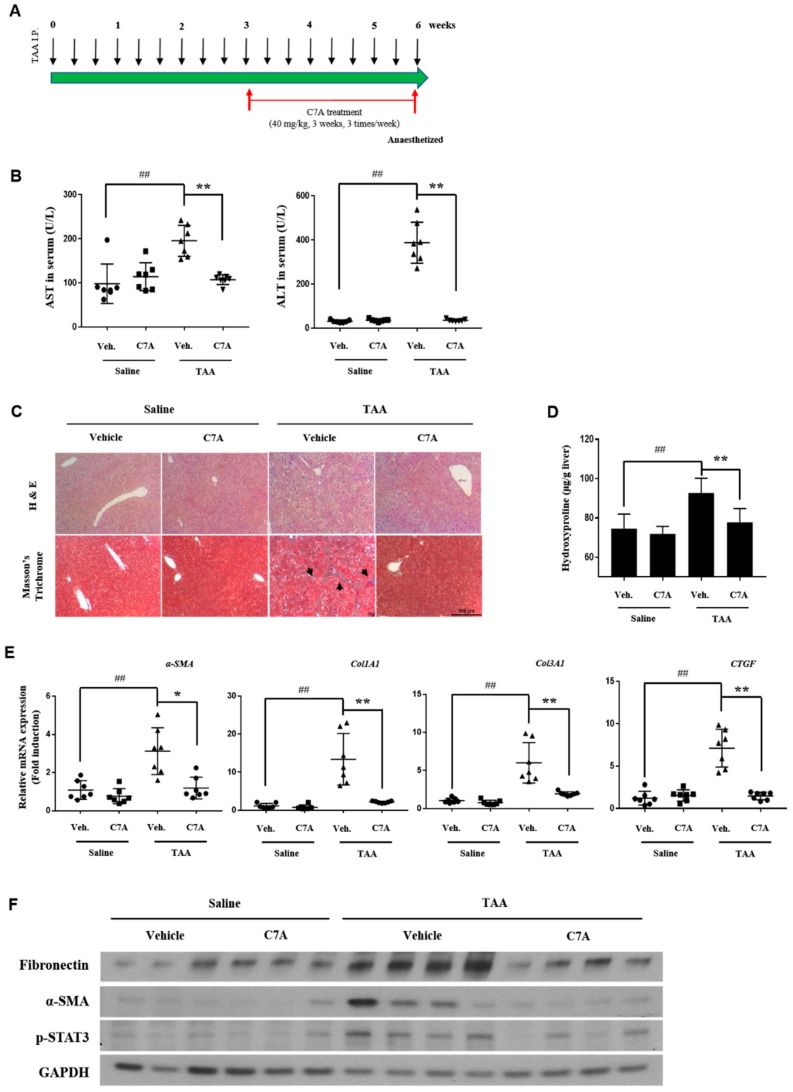
C7A attenuated thioacetamide (TAA)-induced chronic liver fibrosis. (**A**) Mice were given intraperitoneal injections for three weeks of saline or C7A (40 mg/kg) after 3 weeks of saline or TAA (150 mg/kg) treatment. (**B**) Serum aspartate aminotransferase (AST) and alanine transaminase (ALT)levels were analyzed. (**C**) Representative histology section of liver tissues stained with H&E and Masson’s trichrome. (black arrow: collagen deposition; scale bar = 500 μm). (**D**) Liver hydroxyproline contents per gram of liver tissue of mice from each group. (**E**) Gene expressions of *α-SMA, Col1A1, Col3A1,* and *CTGF* were measured by qPCR analysis. (**F**) Western blotting of fibronectin, α-SMA, p-STAT3, and GAPDH in the livers of mice from each group. Each experiment was repeated three times, and values represent mean ± S.D. (n = 7) ## *p* < 0.01 compared with control, * *p* < 0.05, ** *p* < 0.01 compared with TAA treatment group.

To examine the effects of C7A on liver fibrosis, we conducted experiments with a TAA-induced liver fibrosis model. As shown in Figure 6A, C7A (40 mg/kg) was injected I.P. for 3 weeks after 3 weeks of TAA I.P. administration. Blood was collected for aspartate aminotransferase (AST) and alanine transaminase (ALT) analyses at the end of the study. In this study, both AST and ALT levels were significantly increased by TAA treatment, while the levels were strongly decreased by C7A treatment (Figure 6B). H&E and Masson’s trichrome staining showed a clear reduction of collagen deposition (Figure 6C). In addition, the hydroxyproline content was significantly lower in the C7A + TAA group compared to saline + TAA group (Figure 6D). The mRNA levels of fibrotic markers *α-SMA, Col1A1, Col 3A1*, and *CTGF* were significantly lower in the C7A + TAA group than saline + TAA group (Figure 6E). Furthermore, the protein levels of fibrotic markers including fibronectin, α-SMA, and, especially, the phosphorylated STAT3 level were strongly down-regulated in the C7A + TAA group compared to the saline + TAA group (Figure 6F and Table 2).

## 4. Discussion

Hepatic fibrosis is characterized by the abnormal deposition of extracellular matrix (ECM) proteins resulting from continuous liver injury and the wound-healing process [4,46]. Excessive deposition of ECM changes the normal liver architecture to an abnormal structure, which can result in multiple liver function abnormalities and hepatic fibrosis [34]. During liver fibrogenesis, hepatic stellate cells (HSCs) turn to activate and play an important role by enhancing ECM deposition [47,48]. Herein, we elucidated the antifibrotic effects of C7A, isolated from *U. davidiana* var. *japonica*, in transforming growth factor beta (TGF-β1)-induced activated HSCs and thioacetamide (TAA)-induced liver fibrosis in a mice model. C7A is one of the catechin derivatives abundant in green tea and fruits; however, pharmacologic activities of C7A were never reported except antioxidant activities [49]. This study is the first to show the antifibrotic effects as well as mechanism of action of C7A in terms of HSC activation. Moreover, C7A treatment significantly decreased the expression of fibrotic markers including collagen. Our results demonstrate that C7A can be utilized as a potential therapy for the treatment of hepatic fibrosis by inhibiting HSC activation.

HSCs have been considered as key cells in liver fibrogenesis. They have lipid droplets that store vitamin A in the cell body in a quiescent state; however, epithelial cell injuries or hepatitis virus infection transdifferentiate quiescent HSCs into the myofibroblast-like cells that have proliferative, migratory, and contractile properties [48]. When the liver is damaged, hepatocytes, Kupffer cells, endothelial cells, and activated HSCs themselves secrete cytokines that can interact with HSCs [50]. Platelet-derived growth factor (PDGF) and TGF-β1 are the most well-characterized cytokines to induce HSCs. So, we used TGF-β1 to activate HSCs and to up-regulate fibrotic markers, and collagen synthesis demonstrated that TGF-β1 treatment was enough to activate HSCs. Since activated HSCs increase ECM deposition as well as proinflammatory cytokines, they were considered as target cells for hepatic fibrosis therapy [51]. Several clinical trials attempting to develop antifibrotic drugs for hepatic fibrosis by targeting activated HSCs are strong evidence to support this strategy [47]. In this study, we found that the EtOH extract of *U. davidiana* var. *japonica* inhibited HSC activation. Especially, C7A, which was isolated from the EtOAc fraction of the EtOH extract, showed strong anti-fibrotic effects and collagen synthesis in activated HSCs. In addition, C7A treatment significantly decreased fibrotic markers, such as α–SMA, CTGF, and fibronectin as well as collagen deposition, which were induced by TAA treatment. The TAA-induced liver fibrosis model that we used in this study is known to be one of the representative liver fibrosis/cirrhosis models, and the model has a close resemblance to characteristics of human liver cirrhosis [52,53].

STAT3 is an important transcription factor to control cell proliferation and apoptosis of HSCs [13,17]. In response to various growth factors and cytokines, JAK-mediated phosphorylation at tyrosine induces the STAT3 monomer to a heterodimer and translocates to the nucleus, which can activate downstream of STAT3 signaling pathway, including matrix metalloproteinases (MMPs) and connective tissue growth factor (CTGF) [54,55]. These overall procedures of STAT3 activation are responsible for ECM deposition, migration, and apoptosis in HSC and diverse liver disease. Not only inflammatory cytokines like interlukin-6, but TGF-β1 also can activate STAT3 both directly through JAK1 stimulation and indirectly through the SMAD3 pathway [11,56]. In our results, TGF-β1 significantly increased STAT3 phosphorylation and p-STAT3 translocation to the nucleus. Moreover, TGF-β1 increased the expressions of MMP-2, MMP-9, TIMP-2, and CTGF, which are downstream of the STAT3 signaling pathway. We showed that C7A significantly suppressed TGF-β1-induced STAT3 phosphorylation.

Interleukin-6, -8, and -1β are well-identified cytokines to promote the STAT3 signaling pathway [57,58,59]. After TGF-β1 treatment in LX-2 cells, expressions of interleukin-6, -8, and -1β were significantly increased in the mRNA level; however, down-regulation of interleukins was not observed by C7A treatment (data not shown). Instead, C7A suppressed translocation of p-STAT3 to the nucleus, which can be observed by previously reported STAT3 inhibitors such as sorafenib and HJC0123 in HSCs [11,14]. In addition, we measured the effects of C7A on STAT3 downstream genes *MMP-2* and *MMP-9*. MMP-2 and MMP-9 are gelatinases that can degrade the ECM and play important roles in cell migration and proliferation of HSCs [60]. They are expressed during HSC activation after liver injury. They are considered the putative target of STAT3, and high expressions were observed in hepatic fibrosis patients, liver fibrosis animal models, and activated HSCs [61]. This study demonstrated that MMP-2 and MMP-9 were increased in TGF-β1-activated HSCs, and C7A significantly inhibited its expression, which imply that C7A potentially reduces cell migration and proliferation of activated HSCs.

## 5. Conclusions

Our study demonstrates that four catechins, **1**–**4**, identified from *U. davidiana* var. *japonica* showed an inhibitory effect on HSC activation. Among them, (–)-catechin-7-*O*-β-d-apiofuranoside (**1;** C7A) showed the most potent effects on collagen synthesis in HSCs. In our results, C7A strongly inhibited TGF-β1-induced fibrotic responses such as expression of fibronectin, α-SMA, and CTGF. We also elucidated that C7A suppressed the STAT3 signaling pathway, which is an important signal in HSC activation. Furthermore, C7A significantly inhibited ECM deposition in the TAA-induced liver fibrosis mice model. Importantly, this is the first study to report the hepatic protective effects of C7A via inhibition of HSC activation. Our findings provide experimental evidence on the molecular explanation of C7A for the inhibition of HSC activation in vivo and in vitro. Our study suggests that C7A can be a potential candidate as a therapeutic agent in hepatic fibrosis.

## Figures and Tables

**Table 1:** Lists of qPCR primers.

Gene	Species	Forward	Reverse
*α-SMA*	Human	CTGGCATCGTGCTGGACTCT	GATCTCGGCCAGCCAGATC
*CTGF*	Human	GGCTTACCGACTGGAAGAC	AGGAGGCGTTGTCATTGG
*Fibronectin*	Human	CAGTGGGAGACCTCGAGAAG	TCCCTCGGAACATCAGAAAC
*MMP-9*	Human	TTTGACAGCGACAAGAAGTGG	GGGCGAGGACCATAGAGG
*MMP-2*	Human	GAGAACCAAAGTCTGAAGAG	GGAGTGAGAATGCTGATTAG
*Col 1A1*	Human	GGCAACAGCCGCTTCACCTAC	GCGGGAGGACTTGGTGGTTTT
*TIMP-1*	Human	TTGACTTCTGGTGTCCCCAC	GCTTCTGGCATCCTGTTGTT
*TIMP-2*	Human	ACAGGCGTTTTGCAATGCA	GGGTTGCCATAAATGTCGTTTC
*α-SMA*	Mouse	GTTCAGTGGTGCCTCTGTCA	ACTGGGACGACATGGAAAAG
*Col 1A1*	Mouse	TTCGGACTAGACATTGG	GGGTTGTTCGTCTGTTTC
*Col 3A1*	Mouse	ACGTAGATGAATTGGGATGCAG	GGGTTGGGGCAGTCTAGTG
*CTGF*	Mouse	TGACCCCTGCGACCCACA	TACACCGACCCACCGAAGACACAG

**Table 2:** Body and liver weight in saline + saline, C7A + saline, saline + TAA, and C7A + TAA treated groups. Mean ± S.D are shown (n = 7).

	Saline + Saline	C7A + Saline	Saline + TAA	C7A + TAA
**Initial body weight (g)**	24.13 ± 0.93	24.21 ± 1.26	24.41 ± 0.96	24.70 ± 1.46
**Final body weight (g)**	29.61 ± 2.93	27.91 ± 3.50	25.78 ± 1.16	26.25 ± 1.44
**Liver weight (g)**	1.33 ± 0.26	1.23 ± 0.20	1.26 ± 0.30	1.07 ± 0.09
**Liver weight/body weight (×100)**	4.51 ± 0.52	4.39 ± 0.29	4.89 ± 1.11	4.08 ± 0.24
